# Possible Roles of IL-33 in the Innate-Adaptive Immune Crosstalk of Psoriasis Pathogenesis

**DOI:** 10.1155/2019/7158014

**Published:** 2019-10-14

**Authors:** Serafinella Patrizia Cannavò, Lucrezia Bertino, Eleonora Di Salvo, Valeria Papaianni, Elvira Ventura-Spagnolo, Sebastiano Gangemi

**Affiliations:** ^1^Section of Dermatology, Department of Clinical and Experimental Medicine, University of Messina, 98125 Messina, Italy; ^2^National Research Council of Italy (CNR), Institute of Biological Resources and Marine Biotechnologies (IRBIM), Messina, Italy; ^3^National Research Council of Italy (CNR)-Institute of Applied Science and Intelligent System (ISASI), Messina, Italy; ^4^Legal Medicine Section, Department for Health Promotion and Mother-Child Care, University of Palermo, Via del Vespro 129, 90127 Palermo, Italy; ^5^School and Operative Unit of Allergy and Clinical Immunology, Department of Clinical and Experimental Medicine, University of Messina, 98125 Messina, Italy

## Abstract

**Background:**

IL-33 belongs to the IL-1 family, playing a role in several biologic processes as well as in the pathogenesis of different diseases, including skin pathologies. It acts as an alarmin, released by damaged cells. Binding to a ST2 receptor, it stimulates many immune cells such as ILC2 and Th2 cells. IL-33/ST2 axis seems to be involved in Th17 response. According to this, a review was performed to analyze if IL-33 even interplay in the onset of psoriasis, a Th1/Th17 inflammatory disease.

**Methods:**

Data obtained from the included articles are study author name, publication date, group studied, clinical and biological variables, laboratory tests, and outcome of interest of the study.

**Results:**

Data are obtained from the 19 studies identified, which assessed the association between IL-33 and psoriasis.

**Discussion:**

It seems to promote the innate-adaptive immune crosstalk: it could induce mast cells and neutrophil response after being released by injured keratinocytes and after stimulation by some cytokines, in particular TNF*α*, INF*γ*, and IL-17A. In addition, it seems to be involved from the onset of disease to the development of comorbidities, as psoriatic arthritis.

**Conclusion:**

The core of the future research on psoriasis could be to fully understand the role of this complex cytokine, in order also to find a new therapeutic approach.

## 1. Introduction

Interleukin-33 (IL-33) is a member of the IL-1 family, which also includes other 11 members such as IL-1*α*, IL-1*β*, IL-18, IL-36, IL-37, and IL-38. It is constitutively expressed in the nuclei of endothelial cells, epithelial of barrier tissue, and fibroblast-like cells [1].

IL-33 acts in several biological processes, for instance, tissue homeostasis, growth, repair, and immune response. At the same time, it plays a role in the pathogenesis of different diseases, among which infections, asthma, allergic rhinitis, cardiovascular diseases, rheumatoid arthritis, inflammatory bowel disease, diabetes, and various cancers. It is involved in skin diseases, including atopic dermatitis, prurigo, chronic urticaria, vitiligo, and psoriasis (Ps) [2–5].

IL-33 could have a dual function: acting either extracellularly as a cytokine or intracellularly as a nuclear factor regulating gene expression. It is released as a consequence of cell damage or cellular stress. After its release, it acts as an alarmin activating the cells which play a key role in innate-adaptive immunity [6]. It exerts its activity by binding to a receptor complex consisting of its primary receptor, ST2, and IL-1 receptor accessory protein, IL-1RAcP. There are two forms of ST2: a transmembrane one and a secreted soluble form (sST2), which acts as a decoy receptor [7].

ST2 is expressed by many immune cells involved in type 2 immune response, such as group 2 innate lymphoid cells (ILC2), mast cells, Th2 cells, eosinophils, basophils, and dendritic cells (DC) [8]. After IL-33/ST2 receptor binding, ILC2 are stimulated to produce other several proinflammatory cytokines such as IL-4, IL-5, IL-9, and IL-13. Therefore, ILC2 are also involved in type 2 immune response, parasitic infections, and allergic inflammation, like asthma and atopic dermatitis [9–11].

Moreover, in a study analyzing some allergic airway diseases, Vocca et al. demonstrated that the IL-33/ST2 axis is involved not only in the Th2 response but also in the Th17 one [12]. Recent studies, in fact, revealed that IL-33 stimulated type 1 and 17 immunity cells, such as Th1 cells, Th17 cells, CD8^+^cells, B cells, NK cells, neutrophils, macrophages, and NKT cells, as well as Treg cells [8, 13].

Ps is a Th1/Th17 disease, which is defined as chronic skin inflammation, affecting 2-4% of the population. It results from a dysregulated interplay between keratinocytes (KC) and immune cells, inducing a self-perpetuating loop that amplifies the inflammation, leading also to skin hyperproliferation [14]. Additionally, new pieces of evidence pointed out that Ps is a systemic disease, not only a skin disease [15]. Major comorbidities are arthritis psoriasis (PSA), affecting 30% of Ps patients, cardiovascular pathologies, metabolic syndrome, and Crohn's disease [16, 17].

The pathogenesis of Ps is complex, and many mechanisms are still going to be clarified. For sure, it has genetic [18], epigenetic [19, 20], and environmental factors [18]. The crosstalk between immune cells and cytokines has a key role in Ps development. Whereas Ps was initially considered a Th1-mediated disease, a clear role of IL-23/Th17 axis and Th22 cells has been established in recent years. In particular, Th17 is involved in a loop with DC and KC. IL-17, IL-23, and TNF*α* are secreted by the above-cited T cells which initially underwent differentiation from T cells naïve by interaction with DC, IL-12, and IL-23. Various stimuli could activate DC, for example, by endogenous factors normally sequestered intracellularly in KC [21].

IL-33 is constitutively expressed in epithelial cells and after cell injury could be released [22]. Therefore, IL-33 could play a major role in Ps, according to other Th1/Th17 diseases.

Consequently, we decided to investigate how IL-33 behaves in the pathogenesis of Ps, to understand how it interplay in the innate-adaptive immune crosstalk, also in the most important comorbidity, PSA.

## 2. Results

Table 1 resumes the main data obtained from the 19 studies identified, which assessed the association between IL-33 and Ps.

### 2.1. IL-33, from the Releasing by KC to the Activation of the Immune Response

Two studies reported that IL-33 was expressed in proliferating KC. In particular, Meephansan et al. evidenced that the nuclear staining of IL-33 was observed in the proliferating KC of the spinous layer in a skin biopsy of psoriatic plaques [26]. In a following article by the same authors, they found that IL-33 in normal skin is expressed by the endothelial cells, but in psoriatic skin, it is even present in the nucleus of KC, within the suprabasal layer to the stratum spinosum [29].

IL-33 could be released by KC, after skin damage, leading to a cascade of cellular events, as suggested by Suttle et al. By inducing a Koebner reaction, the authors showed that IL-33 nuclear expression in the Koebner-positive patients decreased at days 1 and 3. Otherwise, it increased at day 7, even if the increase was not statistically significant [33].

In a second study, the same authors collected a sequence of skin biopsies at days 0, 1, and 7, after inducing a Koebner reaction in the patients selected for the study. In skin biopsies from Koebner-positive patients, they observed high levels of IL-6, known to be induced by IL-33 in mast cell. In fact, they observed that the number IL-33^+^ cells increased through the days. But they also evidenced that the number of IL-33^+^ cells was increased also at 0 days in Koebner-negative patient [27].

Few studies demonstrated that some cytokines could increase the release of IL-33. In a study on ex vivo full-thickness skin organ cultures and on normal human epidermal sheets, it was demonstrated that the stimulation with TNF*α* increased the IL-33 mRNA expression in Ps skin compared to untreated skin [31].

Investigating normal human epidermal keratinocytes (NHEK), Meephansan et al. even showed that TNF*α* together with INF*γ* induces the expression of IL-33, which in turn induced the suppression of IL-8 action [26]. In another article, the same authors determined that IL-17A seems to upregulate the IL-33 expression in NHEK culture, probably by induction of ERK, p38/MAPK, and JAK/STAT pathways. They showed also that synergism between IL-17A and TNF*α* does not induce IL-33 [29].

Mitsui et al. confirmed the pieces of evidence seen before, namely, serum IL-33 levels correlate with serum TNF*α* levels and IL-33 leads to NHEK secretion of IL-6 and IL-8. They also found in Ps patient that there was no correlation between IL-33 and IL-6, VEGF and CRP. Finally, they showed that IL-33 was significantly higher in PS patient than those healthy controls and that there was no correlation with PASI score [36].

On the contrary, Batista et al. found no differences in the production of IL-33 in lesional and unaffected skin biopsies of psoriatic patients [30]. Also, Sehat et al. demonstrated that whereas IL-36 and IL-37 serum levels were higher in PS patients than HC, IL-33 serum levels were equal to those in HC. Moreover, they found that all these cytokine serum levels positively correlate with PASI score [41].

Once released, IL-33 seems to induce mast cell activation. Suttle et al. evidenced in vitro that at low concentrations of IL-33 could activate mast cells, otherwise at high concentrations reversed the stimulation back [33].

Hueber et al. demonstrated that mice ST2^−/−^ exhibited reduced cutaneous inflammation compared to wild-type mice. However, the injection of IL-33 in mouse ears induced skin inflammation: they found that the response was partially induced by mast cells and neutrophils [24].

The evidence that IL-33 enhanced the mast cells and neutrophil recruitment promoting inflammation was confirmed by Balato et al. They found high levels of IL-33 in skin biopsies but not in serum. In addition, they evidenced that TNF*α* induced the secretion of important mediators of MC recruitment such as IL-6, VEGF, and MCP-1; this effect was reinforced by IL-33 addition in human culture cells. As seen in previous studies, the author showed that TNF*α*, and not IL-17, induces the secretion of IL-33 from HaCat cells; according to their report, it was present in the nucleus, in the cytoplasm, and in the junctions between KC culture cells [25].

Furthermore, Theoharides et al. reported that in human mast cell culture, peptide substance P (SP) induces the gene expression and secretion of VEGF. They also showed that coadministration of IL-33 in this culture significantly increased this effect. The analysis of skin biopsies even suggests that the gene expression of IL-33 along with histidine decarboxylase (HDC), an indicator of mast cell presence, is considerably increased in both affected and unaffected psoriasis skin. Moreover, the immunohistochemistry indicates that IL-33 is associated with endothelial cells, but mostly with the immune cells in affected skin [23].

Lastly, Patruno et al. showed that IL-33 seems to be also an inflammatory pain mediator. They found that its levels in a skin biopsy of Ps patients positively correlates with NRS scale and Pain Qualities Assessment Scale (PQAS) [34].

### 2.2. IL-33 and PSA

There are five articles that analyze the role of IL-33 in the development of PSA. One study conducted on arthritis and Ps mouse models demonstrated that IL-33 was expressed in the synovium of arthritic mice, but it was not required for the development of arthritis. Moreover, the authors showed that there was no difference between IL-33^−/−^ and WT mice in frequencies of Treg, Th1, and Th17 cells in Ps and arthritis models [37].

A study conducted on human osteoclasts culture showed that IL-33 together with IL-17 increased the gene expression of the proosteoclastogenesis factor (FGF-6, IL-16, and OPN). Moreover, IL-33 also seems to modulate the release of antiosteoclastogenic factor release (FGF-4, IL-4, and FGF-7) and mediators of osteoclast precursor (OCP) homing [39].

The other studies are conducted on humans. Li et al. found high serum levels of IL-33 in Ps and PSA patients compared to healthy controls. Furthermore, IL-33 showed no correlation with PASI, radiographic damage score, osteoclastogenesis-related cytokines, and PSA joint activity index (PSAJAI) [38].

Talabot-Ayer et al. assessed IL-33 and its receptor sST2 levels in serum, synovial fluid (SF), and synovium in patients with PSA. IL-33 is not detectable in serum and SF. Instead, there is a strong nuclear expression in endothelial cells of synovium and synovial fibroblast. They also detected high levels of IL-6 in SF, but they do not evidence a correlation between IL-33 and IL-6 levels. They finally compared these levels with those in patients with rheumatoid arthritis, finding higher levels of IL-33 in the serum and SF of PSA [28].

Shen et al. investigated the association of the IL-33/ST2 axis with comorbidities of patients with PSA, in particular with atherosclerosis and osteoporosis. Plasma sST2 levels resulted to be high in PSA patients, and also, sST2 levels were significantly associated with higher cortical porosity and cortical pore volume. Instead, IL-33, which was detected only in 15% of serum PSA patients, was not associated with these markers of disease [35].

### 2.3. Effects of Ps Treatment on IL-33 Levels

Five articles in the review analyzed the effect of therapeutical option for Ps treatment. One study [38] showed no changes in Ps patient serum levels of IL-33 after TNF*α* inhibitor therapy (TNF*α*i). Another one demonstrated that UVBnb phototherapy increased IL-33 levels in serum and skin biopsy of Ps patient. However, in the same articles, Meephansan et al. demonstrated that MTX treatment resulted in decreased skin and blood IL-33 levels [40].

All the others pointed out the reduction of IL-33 levels after anti-TNF*α* treatment. Balato et al. investigated whether adalimumab downregulates IL-33 mRNA expression in skin biopsies of psoriatic patients. The laboratory analysis revealed that this therapy significantly reduces its expression [31].

Also, Vageli et al. investigated the efficacy of etanercept and infliximab on the downregulation of proinflammatory molecules, in particular IL-33, TLR-2, and TLR-9. They detected in skin patient biopsies before the treatment elevated levels of these molecules compared to ones after 12 weeks of therapy, which exhibits a significant reduction. Additionally, PASI score exhibited a positive correlation with the before/after treatment mRNA expression ratio of TLR-2 and TLR-9, but there was no correlation between PASI score and IL-33 mRNA expression [32].

The last article highlighted the reduction of serum IL-33 levels in 10 patients, five Ps, and five PSA, after treatment with adalimumab or infliximab [36].

## 3. Discussion

IL-33 is a cytokine constitutively expressed in endothelial and epithelial cells, which play a role in health and disease. It is involved in many inflammation-related diseases, both Th2 and Th1/Th17 response [1, 42]. In particular, it seems to be involved in the pathogenesis of Ps, which is a Th1/Th17 inflammatory disease.

In fact, the first data pointed out by this review is that IL-33 concentration is always higher in PS patient than HC [23, 24, 26], [29, 31, 34, 36, 39, 41]. Furthermore, its results are expressed in KC and more specifically within the suprabasal layer to the stratum spinosum [26, 29]: it could be linked to the actively proliferating KC which contributes to epidermal hyperplasia, the main histological characteristic of psoriatic lesions. Instead, there are contrasting data on IL-33 serum levels in Ps patients: in two studies are not detectable, in one are higher than in HC while in another are equal to HC [25, 28, 36, 41]. Maybe the differences could be due to an ELISA kit used and to the severity of disease at the moment of the blood sample, which seems to be different from the analysis of material and methods. However, the majority of results suggest that in Ps there is a localized, not generalized inflammatory pattern.

Usually, endothelial and epithelial cells rapidly released IL-33 after tissue injury: the same mechanism could be involved in the pathogenesis of psoriasis [43]. Suttle et al. demonstrated that psoriatic patients with positive Koebner response showed a decrease in epidermal thickness and a transient reduction in epidermal IL-33 immunostaining after tape-stripping. In addition, their experiments performed on KC showed that IL-33 is activated and released by these cells after the damage [33]. Interestingly, in another study concerning Ps, IL-33 was detected not only in the nucleus and in the cytoplasm but also in the junctions between KC [25].

Once released in the microenvironment, IL-33 could act as an alarmin, triggering the cells involved both in the innate immune response, such as mast cells and neutrophils, and in the Th1/Th17 response [39]. Indeed, mast cells can activate some cells of the immune system like eosinophils and neutrophils [27, 28] and can attract KC. These cellular interactions are crucial for the development of skin inflammation, such as in the psoriatic lesion. Once mast cells were considered as “allergic cells,” mast cells are now known to play an important role in the immunopathological mechanisms of Ps [27, 44]. Since gene expression of IL-33 and of HDC, a marker of the mast cells, is significantly increased in psoriatic patients' skin [24], it could be supposed that IL-33 is involved in mast cell recruitment. Maybe the released IL-33 could diffuse from epidermis to dermis triggering ST2^+^ cell, including mast cells. According to this, some studies have demonstrated that IL-33 is a potent activator of mast cells [45, 46]: it induces degranulation and production of various cytokines, including IL-1, IL-6, IL-13, and TNF*α* [25]. One study showed that there was no correlation between IL-33 and IL-6. This data could be in contrast to the statements above, but the authors of the study associated the discrepancy to a different ELISA kit used [36].

An interesting data highlighted by this review is that low concentrations of IL-33 could activate mast cells and high ones downregulated these cells, revealing a dual role of IL-33 [33]. This paradoxical phenomenon is an example of a dose-dependent effect that could be observed in many different biological processes.

Therefore, IL-33 resulted to promote the inflammatory response; in fact, IL-33 promotes angiogenesis and in turn stimulates the recruitment of immune cells to the site of inflammation. One study suggested that IL-33 and a neuropeptide, SP, endorse the release of VEGF from mast cells [23]. This data is very relevant because VEGF is implicated in the pathogenesis of Ps and it correlates with Ps clinical severity.

Interestingly, TNF*α*, INF*γ*, and IL-17A, which are the main effector of the Th1/Th17 response in psoriasis pathogenesis, also resulted to stimulate the IL-33 release [26, 29, 36]. IL-17A seems to upregulate IL-33 expression in NHEK, maybe by induction of ERK, p38/MAPK, and JAK/STAT pathways [22]. Another study showed that these pathways are also implicated in the activation of mast cells, mediated by IL-33 [47]. These data pointed out one more time that IL-33 is fundamental in the innate-adaptive immune crosstalk.

IL-33 augmented levels were found both in skin lesions, as a consequence of KC release, and in unaffected skin biopsies of psoriatic patients, maybe due to the stimulating role of the involved cytokines, such as IL-17A, IL-22, IL-23, TNF*α*, and INF*γ* [30].

Only one study showed that IL-17 could not induce IL-33 expression: although this data resulted being in contrast with the previous statement, this discrepancy could be explained by the usage of a different cell line (HaCat cells) [25].

The importance of TNF*α*, INF*γ*, and IL-17A is also suggested by the effects of Ps treatment on IL-33 levels. TNF*α*I, such as adalimumab, etanercept, and infliximab, are a treatment option for the severe form of the psoriatic disease [48]. They downregulate Th17 response, inhibiting TNF*α* and IL-17, and as a consequence, they may reduce IL-33 levels. In particular, in three papers, IL-33 levels decreased in skin biopsy after TNF*α*I therapy [31, 32, 36]. Besides, in one study, the levels resulted a decrease after the therapy along with TLR-2 and TLR-9, which are other proinflammatory molecules essential in the innate immune response [32]. This data highlighted another time the proinflammatory function of IL-33. MTX is another treatment option for a moderate to severe form of psoriasis which does not respond to topical therapy. It indirectly suppresses IL-17, TNF*α*, and INF*γ*. Patients treated with MTX showed decreased levels of IL-33 [40]. A recent article confirms the efficacy of TNF*α*I plus MTX in reducing IL-33 levels in psoriatic patients [49]. First of all, they found that IL-33 levels are higher than healthy controls, as seen in the above-mentioned articles. Secondly, the levels decrease after therapy with TNF*α*I alone or plus MTX. However, they observed that there was no difference in level reduction between monotherapy and combination therapy. This is perhaps observed because both therapies do not target IL-33. They only cause indirect reduction of IL-33 levels acting on various pathways.

UVBnb phototherapy is the only exception in this context. It was speculated that this therapy provoked increased serum and skin IL-33 levels after the treatment. Other studies confirmed this effect of UVBnb to IL-33 levels in normal skin [50, 51]. In these cases, the authors speculated that IL-33 skin increase was not related to psoriasis development.

Additionally, there are new pieces of evidence that IL-33 induces the expression of TGF-1, Foxp3+, and Treg cells. Bruhs et al. speculated that IL-33 was vital for the downregulation of skin inflammation during the induction of contact hypersensitivity (CHS). They also reported that this inhibitory stripping effect could be reverted by an anti-IL-33-antibody treatment. On the other hand, exogenous IL-33 repressed the induction of CHS and stimulated Treg [52]. This once again underlines the pleiotropic role of IL-33 and the central involvement of the immune system in Ps.

Genetic studies discovered that IL-33 leads to the growth of IL-10+ Tregs in the skin during the change from acute to chronic inflammation; this transition could suppress type 1 immunity and stimulate the development of a protumorigenic type 2 skin inflammation [53]. In fact, IL-33+ cells and Tregs are considerably and specifically augmented in precancerous chronic inflammatory diseases of skin and bowel in humans [54]. A recent study reported that the IL-33/Treg axis could also play a pivotal role in skin cancer progress in other cancer-prone inflammatory diseases of the skin. However, it is critical to highlight the polyvalent IL-33 function and its dual effects on carcinogenesis. While some researches demonstrate that IL-33/Treg axis has favouring effects on cancer, IL-33 signaling onto CD8+ T cells and NKs can be antitumorigenic [55].

This review supports that IL-33 does not only interplay in the development of Ps but also it is involved in the pathogenesis of comorbidities. High levels of IL-33 was found higher in PSA patient than HC [28, 35, 36, 38]. In particular, one study evidenced that IL-33 is detectable in the synovia but not in serum and in synovial fluid [28], data confirmed also by a second study in which plasma levels are detectable only in 15% PSA patients, maybe because it forms complex with sST2, which is found in high concentration in plasma of PSA patients [35].

More in detail, IL-33 seems to be involved in bone remodeling, promoting the innate immunity response where it has an important role. In fact, it was demonstrated that IL-33, along with OPN, IL-17, and TNF-*α*, induced the release of proosteogenic factors from the skin, i.e., RANKL, which induces OCP differentiation from monocytes. The resorptive function was validated also by the result of one study in which sST2 significantly correlated with higher cortical porosity and cortical pore volume in PSA patients, underlying IL33/ST2 axis is linked with osteoporosis in PSA [35].

Some studies suggested that it has also, in this case, a dual role: it has not only a proosteogenic action but also an anticlastogenic one [39, 56, 57]. The importance of this aspect is confirmed by the radiographical osteoarticular presentation of PSA in which coexist the aspects of appositional and resorptive activity [58].

In contrast with the statements evidenced in this review, results showed by one study conducted on a mouse model of arthritis and psoriasis showed that IL-33 does not affect Th1, Th17, and Treg response in the pathogenesis of Ps and PSA, suggesting the idea that it is not crucial for chronic inflammation development [37]. However, it was found that mice and humans show strong differences in epidermal expression and regulation of IL-33. This supports the essential concept that mouse models are not always appropriate to clarify IL-33 role in the skin [59].

The last interesting evidence pointed out by this review is that the IL-33 level seems not being correlated to disease severity score, except in one case [41]: it was not found a correlation between IL-33 and PASI score [27, 32], PaSAJA and radiographic damage score [38]. This could reflect that IL-33 levels match to an increased systemic inflammatory status. Nevertheless, one study pointed out that IL-33 levels positively correlated with NRS scale and PQAS. Therefore, IL-33 could be also a pain mediator involved in psoriatic nociception [34].

**Figure 1:**
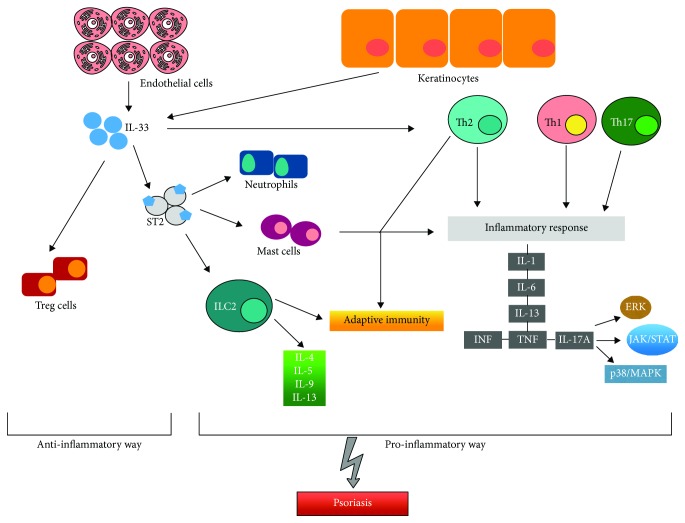
Inflammatory and immunological pathways activated by IL-33 causing the onset of psoriasis.

IL-33 was always described as a cytokine that induces Th2 response, as it was suggested by many papers of its involvement in allergic skin disease and atopic dermatitis. However, in this review, IL-33 appears increasingly involved in the pathogenesis of Ps, especially in the crosstalk between innate and adaptive immune response. It seems to behave in a pleiotropic way according to the stimulus applied, sometimes acting in a proinflammatory way, sometimes playing an anti-inflammatory role. It is involved also in the development of Ps comorbidities and in psoriatic nociception (Figure 1).

In conclusion, our studies showed that IL-33 is a very complex interleukin because of its multiple roles (Figure 1). According to its isoform or its collocation intra- or extracellular, it could facilitate a Th1 or a Th2 response. This balance, pending in a direction or in another could generate a protective or damaging function. As in Ps or cancer-prone cases, this alarmin acts as a detrimental, main, player. For this reason, further studies should focus on all the possible immune activation pathways of IL-33 in order to piece together its pathogenetic role in many chronic inflammatory diseases. Understanding this complex cytokine, its processing, secretion, regulation, and function could be the core of the future research in the field of Ps, in order also to find a new therapeutic approach. There are promising results about anti-IL-33 antibody efficacy in inflammatory diseases. ST2 and IL-33 blocking antibodies showed to be effective in asthma and atopic dermatitis [43, 60]. Furthermore, some studies have reported that these antibodies had an analgesic role, as in arthritis pain [61]. However, experimental evidences in PS are currently lacking.

## 4. Materials and Methods

This literature review was conducted employing PubMed database. On this website, we searched for articles from inception to November 24, 2018 using key terms related to Ps, “psoriasis”, and to IL-33, “interleukin 33” and “IL-33”.

We read the abstracts of those articles which titles suggested they analyzed the association between IL-33 and psoriasis. The entire article was read only if the abstract indicated that the article potentially met the inclusion criteria. Finally, we reviewed and searched the references of these articles in order to identify further studies which could be included. The inclusion criteria were English language and research papers. Articles were excluded by title, abstract, and full text for irrelevance to the topic in question.

Data obtained from the articles included study author name, publication date, group studied, clinical and biological variables, laboratory tests, and outcome of interest of the study.

## Figures and Tables

**Table 1:** Data obtained by the studies included in the review. For each study, the table reports the species examined (animals, culture cells, or humans), the number of patients included in research, and the type of tissue sample analyzed to detect IL-33 concentration. It shows if IL-33 concentration is higher, lower, or equal with respect to health controls. The table also includes other detected cytokines in the study, the correlation between IL-33 concentration and severity score disease in case it was analyzed, and if therapy modified IL-33 levels.

Author, year	Animals	Cells	Humans	N°Pt	Tissue sample	IL-33 concentration	Severity disease score	Therapy	Laboratory test
Theoharides et al., 2010 [23]	—	x	x	9Ps 7 HC	Skin	High	—	—	SP, VEGF, HDC
Hueber et al., 2011 [24]	x	—	x	5Ps 5HC	Skin	High	—	—	ST2, IL-5, IL-13, CXCL1, MCP-1, MPO, INF-*γ*, IL-1*α*, IL-1*β*, IL-2, IL-4, IL-6, IL-10, IL-12/23p40, IL-17, GM-CSF, FGFbasic, IP-10, MIG, MIP-1*α*, TNF-*α*, VEGF
Balato et al., 2012 [25]	—	x	x	/	Skin blood	High in skin No detectable in blood	—	—	ST2, VEGF, MCP-1, IL-6, IL-20
Meephansan et al., 2012 [26]	—	—	x	5 Ps 2 HC	Skin	High	—	—	IL-8
Suttle et al., 2012 [27]	—	—	x	18Ps	Skin (18 pt) Blood (4 pt)	High	No correlation with PASI	—	IL-6
Talabot-Ayer et al., 2012 [28]	—	—	x	9PSA	Blood SF synovia	No detectable in blood and SF High in synovia	—	—	sST2, IL-6
Meephansan et al., 2013 [29]	—	x	x	7Ps 2HC	Skin	High/low	—	—	ST2
Batista et al., 2013 [30]	—	—	x	20Ps	Skin	Equal	—	—	IL-17A, IL-22, TNF-*α*, INF-*γ*, IL-2, IL-21, IL-27
Balato et al., 2014 [31]	—	x	x	20Ps 10HC	Skin	High/low	—	TNF*α*I reduce IL-33 levels	—
Vageli et al., 2015 [32]	—	—	x	17Ps	Skin	High	No correlation with PASI	TNF*α*I reduce IL-33 levels	TLR-2, TLR-9
Suttle et al., 2015 [33]	—	x	x	18Ps	Skin (18 pt) Blood (4 pt)	High	—	—	ST2
Patruno et al., 2015 [34]	—	—	x	12 Ps 3 HC	Skin	High	—	—	—
Shen et al. 2016, [35]	—	—	x	80 PSA	Blood	High in 15%-85%	No correlation with atherosclerosis and BMD	—	sST2
Mitsui et al., 2016 [36]	—	—	x	22Ps 9PSA 17HC	Blood	High in Ps PSA	—	TNF*α*I reduce IL-33	TNF-*α*, CRP, VEGF, IL-6, IL-8
Athari et al., 2016 [37]	x	—	—	/	Skin	—	—	—	—
Li et al., 2017 [38]	—	—	x	20Ps 40PSA 20 HC	Blood	High in Ps PSA	No correlation with PASI and PsAJAI	TNF*α*I have no influence	CRP, OPCs, OPG, TNF-*α*, RANKL, IL12/23p40, IL-34, IL-35, IL36, IL37, IL-38
Raimondo et al., 2017 [39]	—	x	x	20Ps 15 HC	Skin	High	—	—	TNF-*α*, INF-*γ*, IL-4, IL-6, IL-10, IL-13, IL-17, RANKL, OPN, OPG
Meephansan et al., 2018 [40]	—	—	x	14Ps	Skin blood	High	—	MTX downregulates IL-33 levels despite UVBnb	—
Sehat et al., 2018 [41]	—		x	47 Ps 47HC	Blood	Equal	Positively correlated with PASI	—	IL-36, IL-37

Ps: psoriasis, PSA: psoriatic arthritis, HC: health controls, SF: synovial fluid, —: no detected/no analyzed/none, BMD: bone mass density, CRP: C-reactive protein, FGFbasic: fibroblast growth factor basic, GM-CSF: granulocyte-macrophage colony-stimulating factor, HDC: histidine decarboxylase, IP-10: interferon gamma-induced protein 10, MCP-1: monocyte chemoattractant protein-1, MIG: monokine induced by gamma-interferon, MPO: myeloperoxidase, MTX: methotrexate, PaSAJA: PSA joint activity index, OCPs: osteoclast precursors, OPG: osteoprotegerin, OPN: osteopontin, RANKL: receptor activator of nuclear factor-*κ*B ligand, SP: peptide substance P, TNF*α*I: anti-TNF*α* treatments, VEGF: vascular endothelial growth factor.

## References

[1] Cayrol C., Girard J. P. (2018). Interleukin-33 (IL-33): a nuclear cytokine from the IL-1 family. *Immunological Reviews*.

[2] Liew F. Y., Girard J. P., Turnquist H. R. (2016). Interleukin-33 in health and disease. *Nature Reviews. Immunology*.

[3] Di Salvo E., Ventura-Spagnolo E., Casciaro M., Navarra M., Gangemi S. (2018). IL-33/IL-31 axis: a potential inflammatory pathway. *Mediators of Inflammation*.

[4] Vaccaro M., Cicero F., Mannucci C. (2016). IL-33 circulating serum levels are increased in patients with non-segmental generalized vitiligo. *Archives of Dermatological Research*.

[5] Balato A., Raimondo A., Balato N., Ayala F., Lembo S. (2016). Interleukin-33: increasing role in dermatological conditions. *Archives of Dermatological Research*.

[6] Cayrol C., Girard J. P. (2014). IL-33: an alarmin cytokine with crucial roles in innate immunity, inflammation and allergy. *Current Opinion in Immunology*.

[7] Griesenauer B., Paczesny S. (2017). The ST2/IL-33 axis in immune cells during inflammatory diseases. *Frontiers in Immunology*.

[8] Lu J., Kang J., Zhang C., Zhang X. (2015). The role of IL-33/ST2L signals in the immune cells. *Immunology Letters*.

[9] Zhu J. (2018). Mysterious ILC2 tissue adaptation. *Nature Immunology*.

[10] Rothenberg M. E., Saito H., Peebles R. S. (2017). Advances in mechanisms of allergic disease in 2016. *The Journal of Allergy and Clinical Immunology*.

[11] Spooner C. J., Lesch J., Yan D. (2013). Specification of type 2 innate lymphocytes by the transcriptional determinant Gfi1. *Nature Immunology*.

[12] Vocca L., di Sano C., Uasuf C. G. (2015). IL-33/ST2 axis controls Th2/IL-31 and Th17 immune response in allergic airway diseases. *Immunobiology*.

[13] Peine M., Marek R. M., Löhning M. (2016). IL-33 in T cell differentiation, function, and immune homeostasis. *Trends in Immunology*.

[14] Boehncke W. H., Schön M. P. (2015). Psoriasis. *Lancet*.

[15] Grozdev I., Korman N., Tsankov N. (2014). Psoriasis as a systemic disease. *Clinics in Dermatology*.

[16] Dattilo G., Imbalzano E., Casale M. (2018). Psoriasis and cardiovascular risk: correlation between psoriasis and cardiovascular functional indices. *Angiology*.

[17] Veale D. J., Fearon U. (2018). The pathogenesis of psoriatic arthritis. *Lancet*.

[18] Chandran V., Raychaudhuri S. P. (2010). Geoepidemiology and environmental factors of psoriasis and psoriatic arthritis. *Journal of Autoimmunity*.

[19] Hawkes J. E., Nguyen G. H., Fujita M. (2016). microRNAs in psoriasis. *The Journal of Investigative Dermatology*.

[20] Mannucci C., Casciaro M., Minciullo P. L., Calapai G., Navarra M., Gangemi S. (2017). Involvement of microRNAs in skin disorders: a literature review. *Allergy and Asthma Proceedings*.

[21] Deng Y., Chang C., Lu Q. (2016). The inflammatory response in psoriasis: a comprehensive review. *Clinical Reviews in Allergy and Immunology*.

[22] Martin N. T., Martin M. U. (2016). Interleukin 33 is a guardian of barriers and a local alarmin. *Nature Immunology*.

[23] Theoharides T. C., Zhang B., Kempuraj D. (2010). IL-33 augments substance P-induced VEGF secretion from human mast cells and is increased in psoriatic skin. *Proceedings of the National Academy of Sciences*.

[24] Hueber A. J., Alves-Filho J. C., Asquith D. L. (2011). IL-33 induces skin inflammation with mast cell and neutrophil activation. *European Journal of Immunology*.

[25] Balato A., Lembo S., Mattii M. (2012). IL-33 is secreted by psoriatic keratinocytes and induces pro-inflammatory cytokines via keratinocyte and mast cell activation. *Experimental Dermatology*.

[26] Meephansan J., Tsuda H., Komine M., Tominaga S., Ohtsuki M. (2012). Regulation of IL-33 expression by IFN-*γ* and tumor necrosis factor-*α* in normal human epidermal keratinocytes. *The Journal of Investigative Dermatology*.

[27] Suttle M.-M., Nilsson G., Snellman E., Harvima I. T. (2012). Experimentally induced psoriatic lesion associates with interleukin (IL)-6 in mast cells and appearance of dermal cells expressing IL-33 and IL-6 receptor. *Clinical and Experimental Immunology*.

[28] Talabot-Ayer D., McKee T., Gindre P. (2012). Distinct serum and synovial fluid interleukin (IL)-33 levels in rheumatoid arthritis, psoriatic arthritis and osteoarthritis. *Joint Bone Spine*.

[29] Meephansan J., Komine M., Tsuda H., Karakawa M., Tominaga S., Ohtsuki M. (2013). Expression of IL-33 in the epidermis: the mechanism of induction by IL-17. *Journal of Dermatological Science*.

[30] Batista M. D., Tincati C., Milush J. M. (2013). CD57 expression and cytokine production by T cells in lesional and unaffected skin from patients with psoriasis. *PLoS One*.

[31] Balato A., di Caprio R., Canta L. (2014). IL-33 is regulated by TNF-*α* in normal and psoriatic skin. *Archives of Dermatological Research*.

[32] Vageli D. P., Exarchou A., Zafiriou E., Doukas P. G., Doukas S., Roussaki-Schulze A. (2015). Effect of TNF-*α* inhibitors on transcriptional levels of pro-inflammatory interleukin-33 and toll-like receptors-2 and -9 in psoriatic plaques. *Experimental and Therapeutic Medicine*.

[33] Suttle M. M., Enoksson M., Zoltowska A., Chatterjee M., Nilsson G., Harvima I. T. (2015). Experimentally induced psoriatic lesions associate with rapid but transient decrease in interleukin-33 immunostaining in epidermis. *Acta Dermato-Venereologica*.

[34] Patruno C., Napolitano M., Balato N. (2015). Psoriasis and skin pain: instrumental and biological evaluations. *Acta Dermato-Venereologica*.

[35] Shen J., Shang Q., Wong C. K. (2016). Carotid plaque and bone density and microarchitecture in psoriatic arthritis: the correlation with soluble ST2. *Scientific Reports*.

[36] Mitsui A., Tada Y., Takahashi T. (2016). Serum IL-33 levels are increased in patients with psoriasis. *Clinical and Experimental Dermatology*.

[37] Athari S. K., Poirier E., Biton J. (2016). Collagen-induced arthritis and imiquimod-induced psoriasis develop independently of interleukin-33. *Arthritis Research & Therapy*.

[38] Li J., Liu L., Rui W. (2017). New interleukins in psoriasis and psoriatic arthritis patients: the possible roles of interleukin-33 to interleukin-38 in disease activities and bone erosions. *Dermatology*.

[39] Raimondo A., Lembo S., di Caprio R. (2017). Psoriatic cutaneous inflammation promotes human monocyte differentiation into active osteoclasts, facilitating bone damage. *European Journal of Immunology*.

[40] Meephansan J., Subpayasarn U., Ponnikorn S. (2018). Methotrexate, but not narrowband ultraviolet B radiation, suppresses interleukin-33 mRNA levels in psoriatic plaques and protein levels in serum of patients with psoriasis. *The Journal of Dermatology*.

[41] Sehat M., Talaei R., Dadgostar E., Nikoueinejad H., Akbari H. (2018). Evaluating serum levels of IL-33, IL-36, IL-37 and gene expression of IL-37 in patients with psoriasis vulgaris. *Iranian Journal of Allergy, Asthma and Immunology*.

[42] Bertheloot D., Latz E. (2017). HMGB1, IL-1*α*, IL-33 and S100 proteins: dual-function alarmins. *Cellular & Molecular Immunology*.

[43] Theoharides T. C., Petra A. I., Taracanova A., Panagiotidou S., Conti P. (2015). Targeting IL-33 in autoimmunity and inflammation. *The Journal of Pharmacology and Experimental Therapeutics*.

[44] Harvima I. T., Nilsson G., Suttle M.-M., Naukkarinen A. (2008). Is there a role for mast cells in psoriasis?. *Archives of Dermatological Research*.

[45] Allakhverdi Z., Smith D. E., Comeau M. R., Delespesse G. (2007). Cutting edge: the ST2 ligand IL-33 potently activates and drives maturation of human mast cells. *Journal of Immunology*.

[46] Iikura M., Suto H., Kajiwara N. (2007). IL-33 can promote survival, adhesion and cytokine production in human mast cells. *Laboratory Investigation*.

[47] Drube S., Kraft F., Dudeck J. (2016). MK2/3 are pivotal for IL-33–induced and mast cell–dependent leukocyte recruitment and the resulting skin inflammation. *The Journal of Immunology*.

[48] Ighani A., Partridge A. C. R., Shear N. H. (2018). Comparison of management guidelines for moderate-to-severe plaque psoriasis: a review of phototherapy, systemic therapies, and biologic agents. *Journal of Cutaneous Medicine and Surgery*.

[49] Yu Q., Tong Y., Cui L. (2019). Efficacy and safety of etanercept combined plus methotrexate and comparison of expression of pro-inflammatory factors expression for the treatment of moderate-to-severe plaque psoriasis. *International Immunopharmacology*.

[50] Byrne S. N., Beaugie C., O'Sullivan C., Leighton S., Halliday G. M. (2011). The immune-modulating cytokine and endogenous alarmin interleukin-33 is upregulated in skin exposed to inflammatory UVB radiation. *The American Journal of Pathology*.

[51] Meephansan J., Komine M., Tsuda H., Tominaga S., Ohtsuki M. (2012). Ultraviolet B irradiation induces the expression of IL-33 mRNA and protein in normal human epidermal keratinocytes. *Journal of Dermatological Science*.

[52] Bruhs A., Proksch E., Schwarz T., Schwarz A. (2018). Disruption of the epidermal barrier induces regulatory T cells via IL-33 in mice. *The Journal of Investigative Dermatology*.

[53] Siede J., Fröhlich A., Datsi A. (2016). IL-33 receptor-expressing regulatory T cells are highly activated, Th2 biased and suppress CD4 T cell proliferation through IL-10 and TGF*β* release. *PLoS One*.

[54] Schiering C., Krausgruber T., Chomka A. (2014). The alarmin IL-33 promotes regulatory T-cell function in the intestine. *Nature*.

[55] Ameri A. H., Moradi Tuchayi S., Zaalberg A. (2019). IL-33/regulatory T cell axis triggers the development of a tumor-promoting immune environment in chronic inflammation. *Proceedings of the National Academy of Sciences*.

[56] Mine Y., Makihira S., Yamaguchi Y., Tanaka H., Nikawa H. (2014). Involvement of ERK and p38 MAPK pathways on interleukin-33-induced RANKL expression in osteoblastic cells. *Cell Biology International*.

[57] Schulze J., Bickert T., Beil F. T. (2011). Interleukin-33 is expressed in differentiated osteoblasts and blocks osteoclast formation from bone marrow precursor cells. *Journal of Bone and Mineral Research*.

[58] Barnas J. L., Ritchlin C. T. (2015). Etiology and pathogenesis of psoriatic arthritis. *Rheumatic Diseases Clinics of North America*.

[59] Sundnes O., Pietka W., Loos T. (2015). Epidermal expression and regulation of interleukin-33 during homeostasis and inflammation: strong species differences. *The Journal of Investigative Dermatology*.

[60] Hernandez-Santana Y. E., Giannoudaki E., Leon G., Lucitt M. B., Walsh P. T. (2019). Current perspectives on the interleukin-1 family as targets for inflammatory disease. *European Journal of Immunology*.

[61] Fattori V., Hohmann M. S. N., Rossaneis A. C. (2017). Targeting IL-33/ST2 signaling: regulation of immune function and analgesia. *Expert Opinion on Therapeutic Targets*.

